# Discovery Potential Hub Genes and Pathways in Keloid Fibroblast Development Based on Bioinformatics Analysis

**DOI:** 10.1155/ijog/2089205

**Published:** 2025-11-21

**Authors:** Boao Zhao, Wei Zhang, Wende Yao, Xinyi Li, Tao Yang, Hui Cheng, Xiaojing Li

**Affiliations:** ^1^ Department of Plastic Surgery, The First Affiliated Hospital of Anhui Medical University, Hefei, Anhui, China, ahmu.edu.cn

**Keywords:** bioinformatics, GEO, hub genes, keloid

## Abstract

**Background:**

Keloid is a common pathological scar tissue, which invades the surrounding normal skin and leads to symptoms such as pain, pruritus, erythema, and edema, thereby impacting the quality of life. In this study, we conducted bioinformatics analysis of keloid fibroblasts and normal skin tissue to identify DEGs and the pathways involved in the mechanism of keloid fibroblast proliferation.

**Methods:**

GSE145725 was downloaded from the Gene Expression Omnibus (GEO) database, including nine keloid fibroblasts and 10 normal tissue fibroblast samples. GSE158395 included four lesional and three nonlesional samples from keloid patients, and six normal skin tissue samples were also evaluated. Through bioinformatics analysis, we established diagnosis model, and at the same time, we predicted therapeutic targets in the DSigDB database.

**Results:**

Six key genes were screened out by bioinformatics analysis, including BMP4, SPP1, HIF1*α*, POSTN, WNT5A, and SMAD3. Subsequently, three of these genes (BMP4, POSTN, and WNT5A) were found to be significantly associated with keloids. Paricalcitol and phosphine were identified as potential therapeutic candidates.

**Conclusions:**

This study identified three hub genes—BMP4, POSTN, and WNT5A—that are closely linked to keloid fibroblast hyperplasia and may serve as potential biomarkers for inhibiting keloid fibroblast hyperplasia. Further molecular and animal studies are needed to fully understand the mechanisms of keloid development.

## 1. Introduction

Keloids are a type of tissue characterized by the abnormal and excessive growth of fibroblasts and collagen deposition, typically arising from skin wounds. They represent a benign fibrous tissue overgrowth that results in skin swelling, often following the abnormal healing of skin wounds [1–4]. Keloid hyperplasia invades adjacent normal skin, leading to numerous adverse effects [5–7], such as disfigurement, pain, erythema, edema, and restricted movement. These symptoms can result in skin dysfunction and cosmetic deformities, thereby imposing both physical and psychological burdens on patients and significantly impacting their quality of life [4, 8]. However, the precise molecular mechanisms underlying keloid scarring remain unclear. Our research aims to identify key genes and the biological pathways involved in keloid fibroblast hyperplasia, with the goal of providing new insights into their pathogenesis and potentially uncovering novel therapeutic targets. The ultimate aim is to identify new therapeutic targets and potential biomarkers, develop innovative strategies for keloid treatment in clinical settings, and provide novel approaches for the prevention of keloid formation.

Bioinformatics technology is primarily a computational approach that involves the analysis and processing of biological data, including genetic information. It encompasses the collection, storage, and interpretation of biological data, as well as the development and application of computational tools, statistical algorithms, and software to acquire, analyze, predict, and interpret biomedical information [9].

This study leverages bioinformatics technology to identify abnormally expressed genes and to screen for new therapeutic targets and potential biomarkers. Through the analysis of gene expression data, our approach can effectively identify differential expression levels, offering a robust methodology and predictive tool for gene expression studies. Gene expression profiling provides a deeper understanding of the genetic, cellular, and molecular changes that occur during keloid growth. This insight can inform the design of therapeutic regimens aimed at selectively intervening in keloid growth, thereby inhibiting their further proliferation.

In recent decades, advancements in computer technologies, particularly in bioinformatics analysis, have significantly enhanced the accessibility of large datasets. Furthermore, improvements in machine learning software and hardware have increasingly drawn attention to early disease diagnosis and the identification of treatment targets. Therefore, we establish a diagnostic model utilizing the keloid fibroblast dataset and subsequently employ the keloid dataset for external validation to enhance the predictive performance of biomarkers. Furthermore, we investigate the mechanisms associated with keloid formation and treatment through pathway enrichment analysis and drug prediction. The series matrix file dataset GSE145725 was downloaded from the GEO database, along with its corresponding platform files. The data were then organized and screened according to specific criteria to identify significantly differentially expressed genes (DEGs). Additionally, the functional annotations of these DEGs were determined through enrichment analysis. Finally, PPI networks were constructed among the DEGs, and hub genes were identified using the cytoHubba plugin. These hub genes were further validated using the GSE158395 dataset. In summary, the study of keloid formation mechanisms using bioinformatics techniques has identified potential biomarkers that enhance our understanding of the keloid growth process and provide important insights for early intervention in clinical management.

## 2. Materials and Methods

### 2.1. Data Source

**Figure 1:**
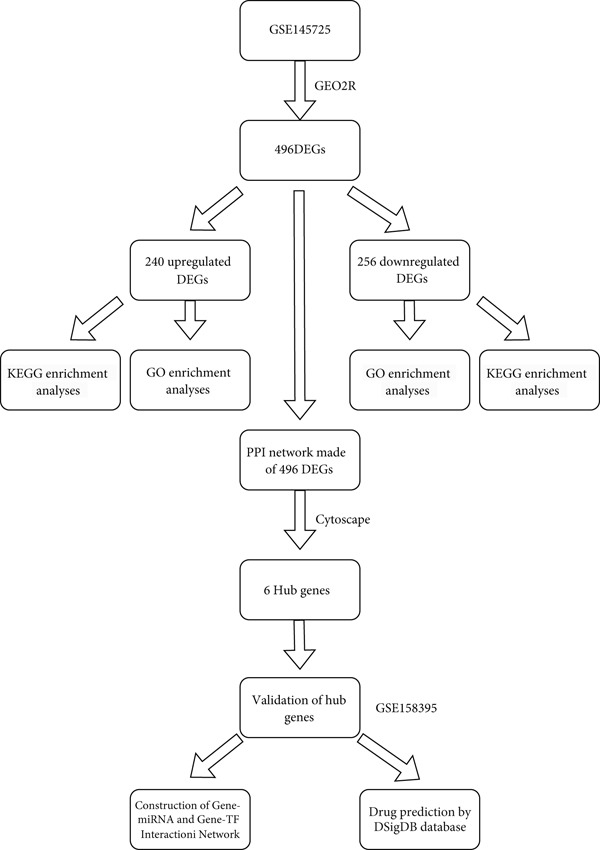
Flow chart of this research.

Figure 1 illustrates the workflow chart of data processing, analysis, and validation. The dataset GSE145725 was downloaded from the GEO database. This dataset was generated using the GPL16043 GeneChip PrimeView Human Gene Expression Array, which includes external spike‐in RNAs. The dataset comprises 19 samples: nine keloid fibroblast samples and 10 normal primary fibroblast samples [10, 11].

### 2.2. Data Processing

The matrix file and platform file of the keloid dataset GSE145725 were obtained from the GEO database. Gene data were identified using GEO2R. The parameters were set as follows: GSM4331585‐GSM4331593 as the keloid group and GSM4331594‐GSM4331603 as the control group. The Benjamini and Hochberg (false discovery rate) method was applied to adjust *p* values. A log transformation was applied to the data with the setting “auto‐detect.” The “apply limma precision weights” option was set to “no,” and “force normalization” was also set to “no.” The “category of platform annotation to display on results” was set to “submitter supplied.” The significance level cut‐off was set to 0.05, and the Log2 fold change (Log2FC) threshold was set to 0, resulting in the exported file named “GSE145725.top.table.tsv.” In Excel, probes were converted, blank rows were removed, and cases where one probe corresponded to multiple genes were handled by retaining only the gene with the highest value for duplicated genes. DEGs were identified using an adjusted *p* value (adj.*p*.Val) < 0.05 and a Log2FC > 1 for upregulated genes and a Log2FC < −1 for downregulated genes.

### 2.3. Enrichment Analysis of Differential Genes

An enrichment analysis of upregulated and downregulated DEGs was conducted using the online software Metascape (http://metascape.org/gp/index.html). GO and KEGG enrichment analyses were performed for both upregulated and downregulated genes. The results were visualized using the clusterProfiler package in R [12–15].

### 2.4. Construction of PPI Networks

PPI network diagrams were constructed using the STRING database. Exploring these relationships can provide insights into the mechanisms of disease occurrence and development. We construct a PPI network after entering DEGs in multiple proteins and selecting *Homo sapiens* in organisms. The minimum required interaction score was set at 0.900, and disconnected nodes in the networks were hidden. Only differentially expressed protein‐coding genes were selected for PPI network construction, as noncoding RNAs do not encode proteins and thus cannot be directly represented in a protein–protein interaction network.

### 2.5. Hub Gene Screening

Cytoscape, a powerful bioinformatics software platform, was utilized to visualize and analyze the complex biological network data [16, 17]. The cytoHubba plugin within Cytoscape was employed to identify hub genes [18]. Five topological analysis methods (degree, radiality centrality, maximum neighborhood component, edge percolated component, and stress centrality) were used to rank and select the Top 10 hub genes.

### 2.6. Validation of Hub Genes

Data from 13 samples (four lesional and three nonlesional samples from keloid patients and six normal skin tissue samples) from the microarray dataset GSE158395 were downloaded from the GEO database (GPL24676 Illumina NovaSeq 6000, *Homo sapiens*). The nonlesional samples were excluded, and the focus was on comparing the differences between normal control samples and lesional samples. The validation of the screened hub genes was then conducted by analyzing the variance among the selected samples [19].

### 2.7. Construction of Gene–miRNA and Gene–Transcription Factor (TF) Interaction Network

NetworkAnalyst was used to create interaction networks between genes, miRNAs, and TFs. The parameters used were as follows: organism specified as *Homo sapiens*, ID type set as official gene symbol, comprehensive experimentally validated miRNA–gene interaction data were collected from TarBase v9.0, and the gene–TF interaction database chosen was JASPAR.

### 2.8. DSigDB Database for Drug–Gene Target Identification

To explore potential therapeutic options for keloids, we utilized the DSigDB database, a comprehensive data repository that contains drugs and their target genes. The DSigDB database is an invaluable tool for identifying and understanding the molecular mechanisms underlying drug actions by mapping drugs to their corresponding gene targets [20].

## 3. Result

### 3.1. Identification of DEGs From the Selected Dataset

**Figure 2:**
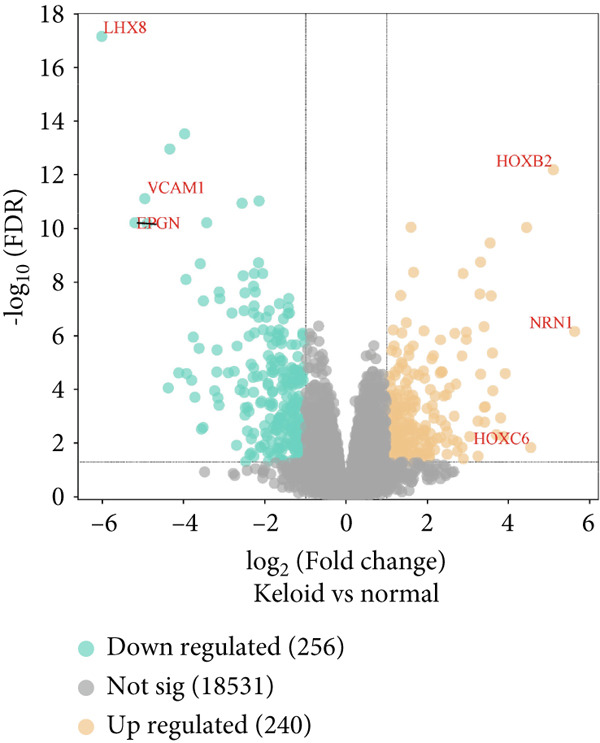
Identification of all DEGs between normal primary fibroblast cell samples compared with the keloid primary fibroblast cell samples.

**Figure 3:**
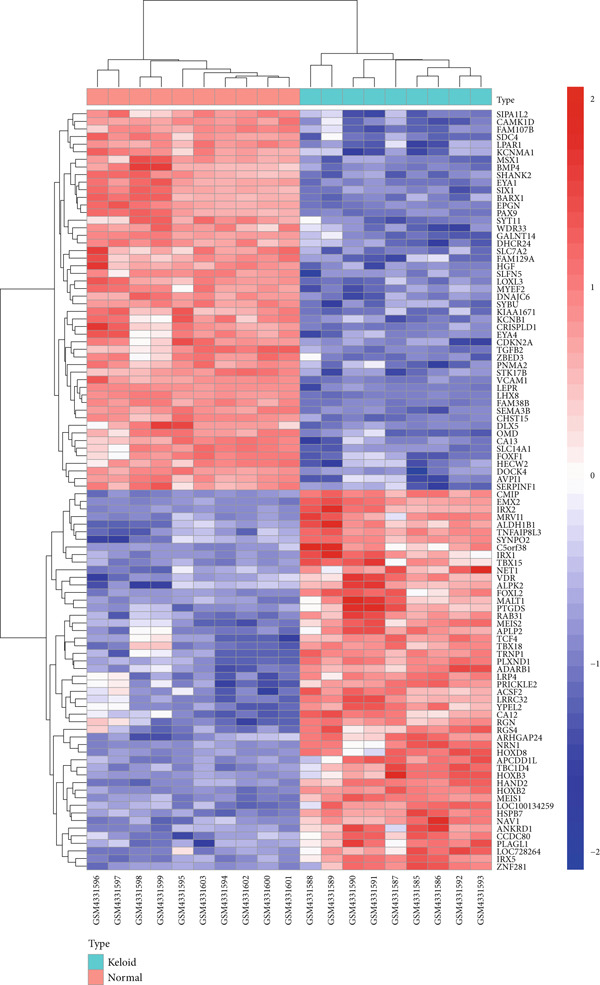
Heatmap shows the expression of genes between normal primary fibroblast cell samples compared with the keloid primary fibroblast cell samples.

The gene expression profile dataset GSE145725 was downloaded from the GEO. A total of 496 DEGs were identified, including 240 upregulated genes and 256 downregulated genes. A volcano plot was generated to visualize the DEGs between normal and keloid primary fibroblast cell samples, with red dots representing upregulated DEGs and green dots representing downregulated DEGs. The Top 3 upregulated genes with the largest difference multiples are HOXB2, NRN1, and HOXC6, while the Top 3 downregulated genes with the largest difference multiples are LHX8, VCAM1, EPGN (Figure 2). For this study, a total of 100 DEGs were selected for detailed analysis, comprising the Top 50 upregulated and Top 50 downregulated genes in the keloid tissue compared to normal skin tissue. As shown in the heatmap (Figure 3), these genes exhibit significant expression differences between keloid and normal tissue sample groups.

### 3.2. GO Functional Enrichment Analysis

**Figure 4:**
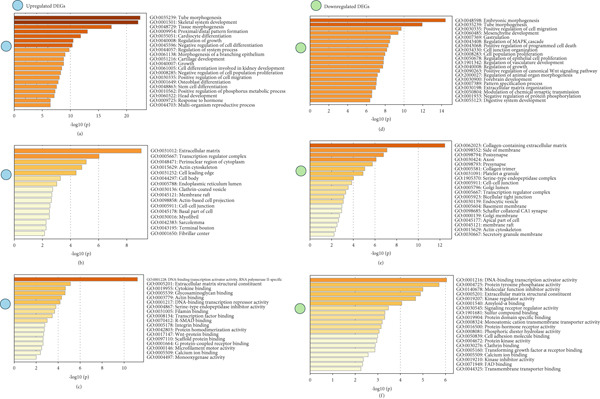
Significant GO terms of 496 DEGs. (a) The biological process of upregulated DEGs. (b) The cellular component of upregulated DEGs. (c) The molecular function of upregulated DEGs. (d) The biological process of downregulated DEGs. (e) The cellular component of downregulated DEGs. (f) The molecular function of downregulated DEGs.

We utilized Metascape, a specialized database resource, to perform GO functional enrichment analysis on all DEGs. The analysis covered three main categories: molecular function (MF), biological process (BP), and cellular component (CC). In BP, the upregulated DEGs were significantly involved in processes such as tube morphogenesis (GO:0035239), skeletal system development (GO:0001501), tissue morphogenesis (GO:0048729), proximal/distal pattern formation (GO:0009954), and cardiocyte differentiation (GO:0035051). The downregulated DEGs were significantly involved in embryonic morphogenesis (GO:0048598), tube morphogenesis (GO:0035239), positive regulation of cell migration (GO:0030335), mesenchyme development (GO:0060485), and gastrulation (GO:0007369). For CC, the results showed that the upregulated DEGs significantly took part in extracellular matrix (ECM) (GO:0031012), transcription regulator complex (GO:0005667), perinuclear region of cytoplasm (GO:0048471), actin cytoskeleton (GO:0015629), and cell leading edge (GO:0031252). Downregulated genes mainly revolved around collagen‐containing ECM (GO:0062023), side of membrane (GO:0098552), postsynapse (GO:0098794), axon (GO:0030424), and presynapse (GO:0098793). In MF, upregulated genes are significantly involved in DNA‐binding transcription activator activity, RNA polymerase II‐specific (GO:0001228), ECM structural constituent (GO:0005201), cytokine binding (GO:0019955), glycosaminoglycan binding (GO:0005539), and actin binding (GO:0003779). For downregulated genes, they mainly take part in DNA‐binding transcription activator activity (GO:0001216), protein tyrosine phosphatase activity (GO:0004725), MF inhibitor activity (GO:0140678), ECM structural constituent (GO:0005201), and kinase regulator activity (GO:0019207). The results are summarized in Figure 4.

### 3.3. KEGG Pathway Analysis

KEGG pathway data were obtained from the DAVID online database (DAVID Functional Annotation Tools). The enrichment analysis was performed using the clusterProfiler package in R. The upregulated DEGs were significantly enriched in cytoskeleton in muscle cells (hsa04820), Wnt signaling pathway (hsa04310), mineral absorption (hsa04978), transcriptional misregulation in cancer (hsa05202), and focal adhesion (FA) (hsa04510), and the downregulated DEGs were significantly enriched in transforming growth factor‐beta (TGF‐*β*) signaling pathway (hsa04350), proteoglycans in cancer (hsa05205), transcriptional misregulation in cancer (hsa05202), hippo signaling pathway (hsa04390), and Wnt signaling pathway (hsa04310). The results of upregulated and downregulated genes can be seen in Figure 5a,b, respectively.

Figure 5Results for KEGG pathway. (a) The KEGG of upregulated DEGs. (b) The KEGG of downregulated DEGs.

**Figure:**
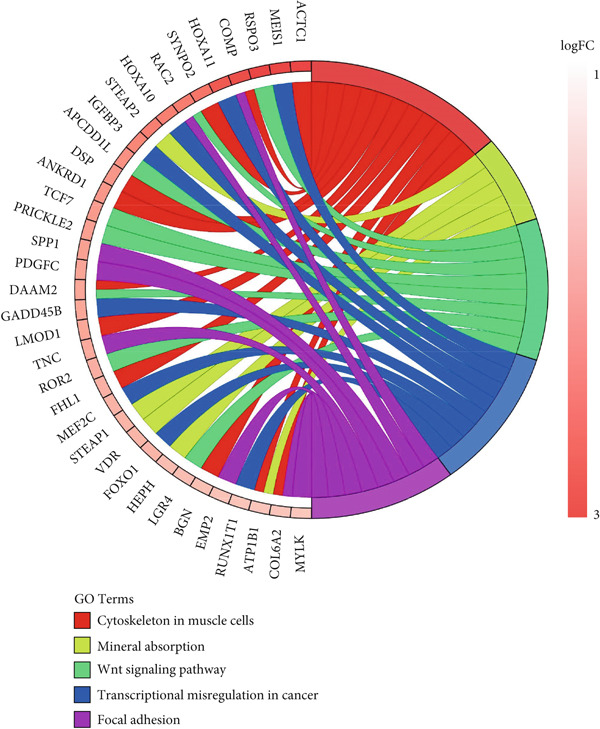
(a)

**Figure:**
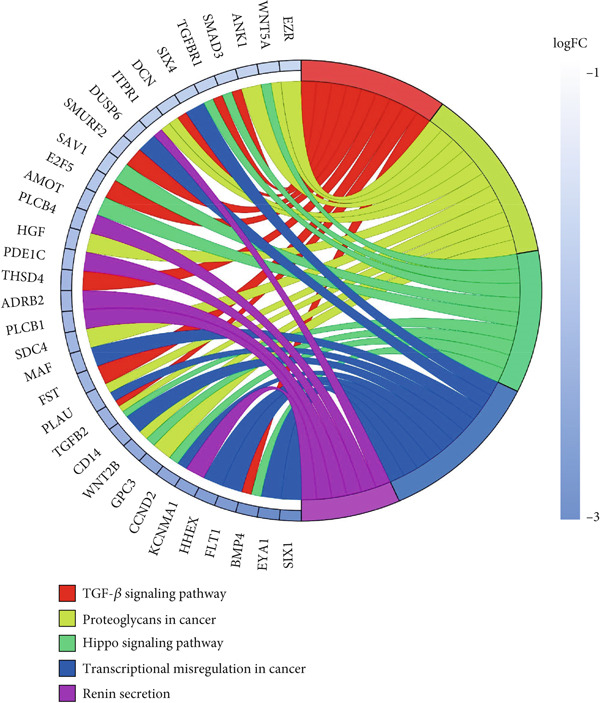
(b)

### 3.4. Construction of PPI Network

**Figure 6:**
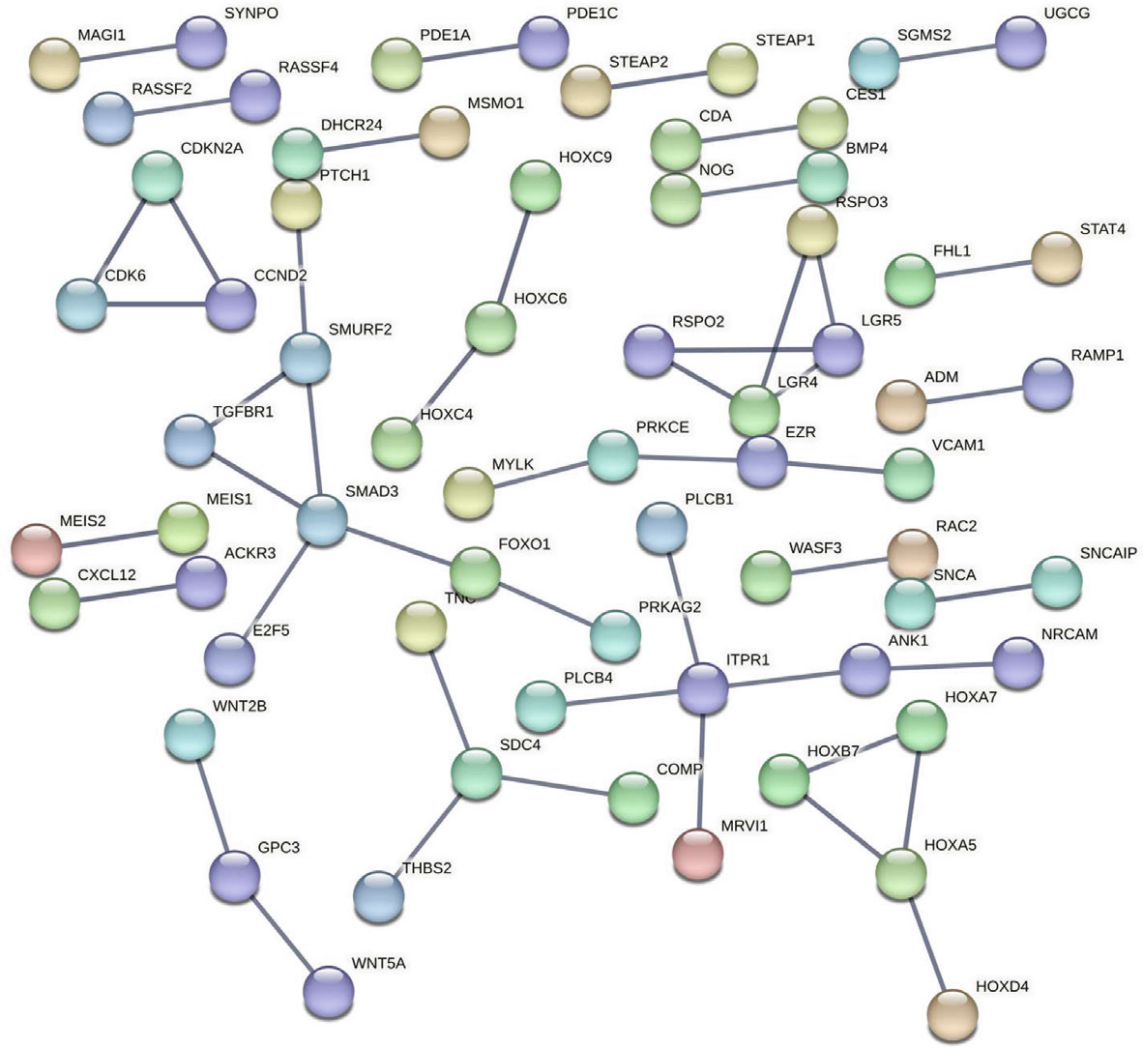
PPI network made of DEGs.

A PPI network was constructed using the STRING database. The resulting network included 492 nodes and 48 edges, where nodes represent DEGs and edges represent the interactions among these DEGs. To identify densely connected regions within the PPI network, we used the MCODE plugin in Cytoscape. The MCODE algorithm helps to detect clusters or modules of highly interconnected proteins, which can provide insights into potential functional subnetworks. Key hub proteins such as SMAD3, BMP4, and WNT5A are centrally positioned, suggesting their potential roles as critical regulators in keloid pathogenesis (Figure 6).

### 3.5. Hub Gene Analysis and Screening

**Figure 8:**
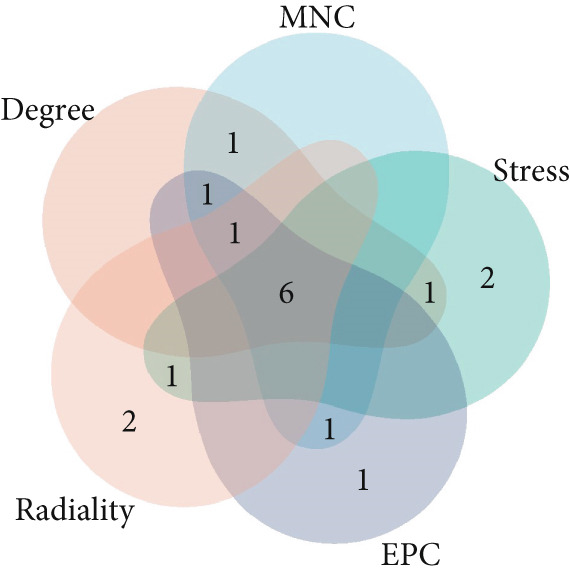
Five topological analysis results by Venn diagram.

To identify and analyze hub genes, we used the cytoHubba plugin in Cytoscape. Five topological analysis methods were employed to screen the Top 10 hub genes (Figure 7). Additionally, a Venn diagram was used to map the overlap of hub genes identified by the five different methods. In the Venn diagram, we get the intersection of five screening methods for a total of six genes: BMP4, SPP1, HIF1*α*, POSTN, WNT5A, and SMAD3 (Figure 8).

Figure 7(a–e) Five topological analysis results by cytoHubba (the redder the color, the higher the rank). (f) PPI network made up of six hub genes.

**Figure:**
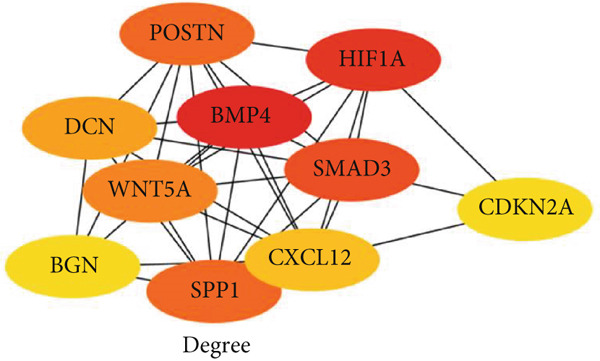
(a)

**Figure:**
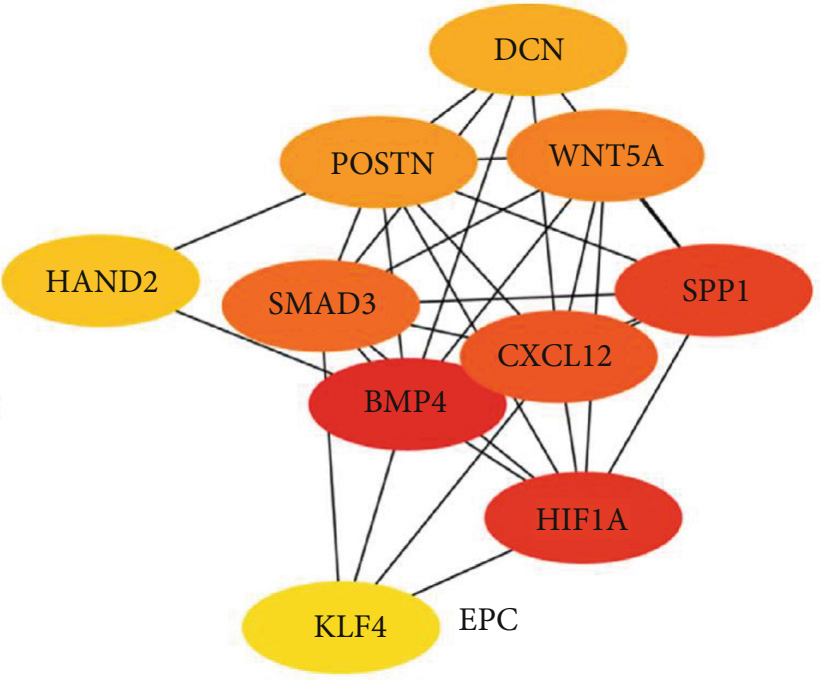
(b)

**Figure:**
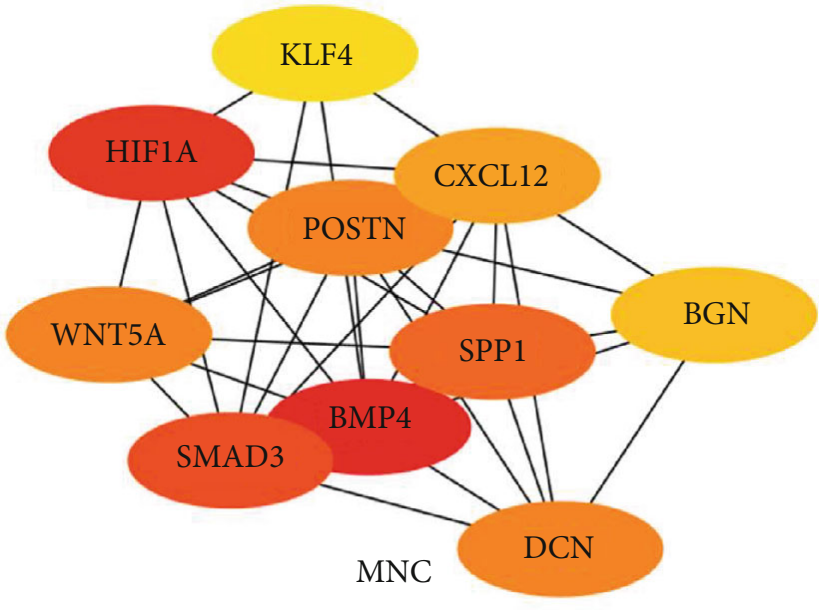
(c)

**Figure:**
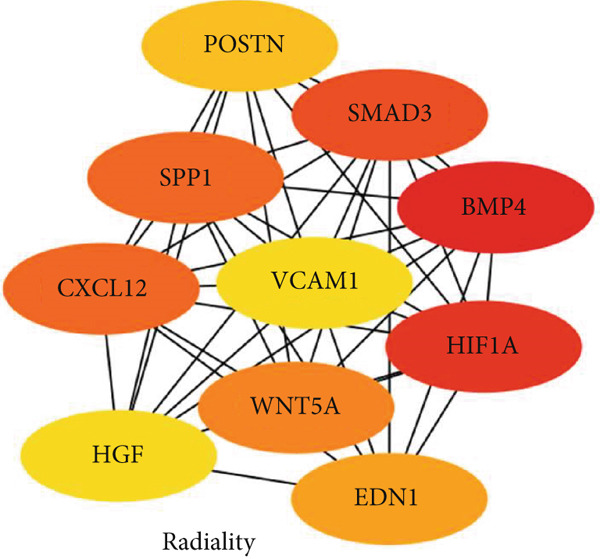
(d)

**Figure:**
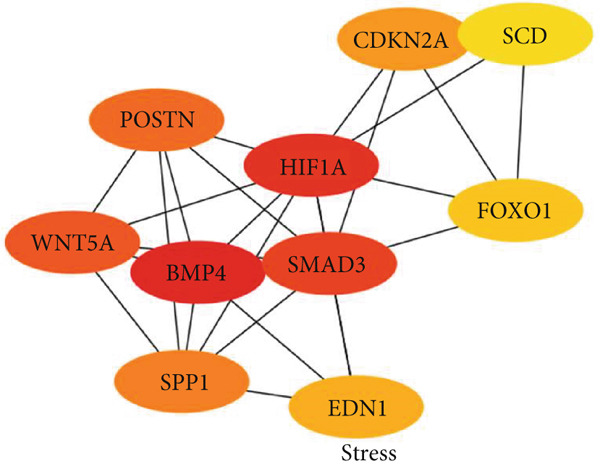
(e)

**Figure:**
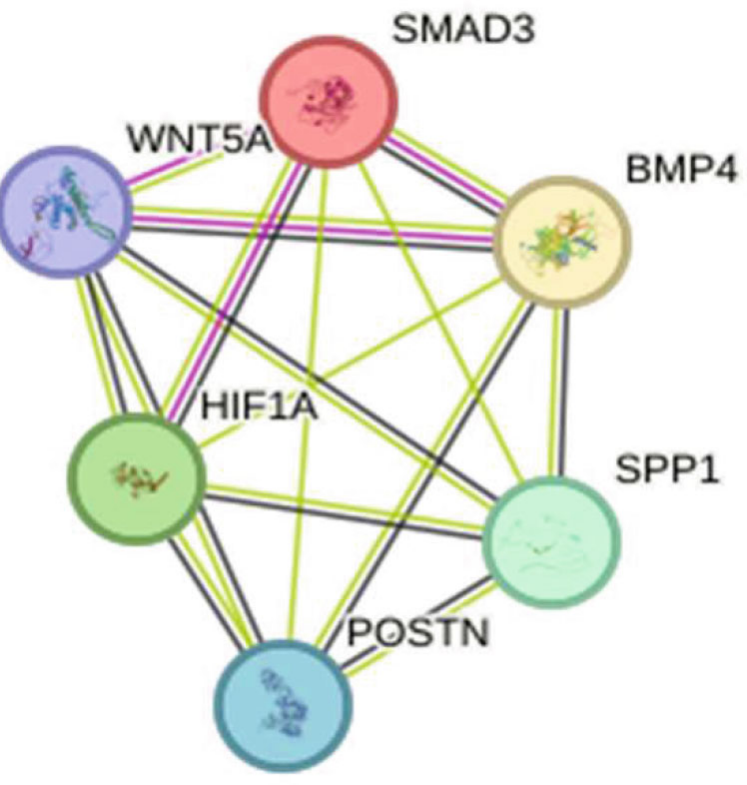
(f)

### 3.6. Validation of Hub Gene Expressions in Keloid Tissue

To validate the expression of the identified six hub genes in keloid fibroblast, we obtained the dataset GSE158395 from the GEO. The validation of the screened hub genes in the GEO dataset revealed that three hub genes—BMP4, POSTN, and WNT5A—were significantly differentially expressed in keloid tissue compared to normal skin tissue. Among them, POSTN was significantly upregulated in keloid samples (*p* < 0.01), consistent with its role in ECM remodeling and fibroblast activation. Similarly, the expression of WNT5A was significantly increased in keloids (*p* < 0.01), suggesting its involvement in noncanonical Wnt signaling and cell migration. In contrast, BMP4 was significantly downregulated (*p* < 0.05), indicating dysregulation of the TGF‐*β*/BMP signaling pathway, which may lead to uncontrolled proliferation of fibroblasts. These findings collectively highlight the molecular characteristics of aberrant signaling pathways surrounding ECM overproduction and the pathogenesis of scarring (Figure 9). Additionally, the diagnostic value of these three hub genes was assessed using ROC (receiver operating characteristic) curve analysis. When three hub genes were utilized as distinct diagnostic indications, the ROC curve data demonstrated that BMP4 in the GSE158395 was 0.958, reflecting its significant downregulation in pathological conditions. POSTN and WNT5A demonstrated perfect discriminative ability with AUC values of 1.000, indicating their high specificity and sensitivity in identifying keloid tissue (Figure 10).

Figure 9(a–f) Expression levels of hub genes in keloid.

**Figure:**
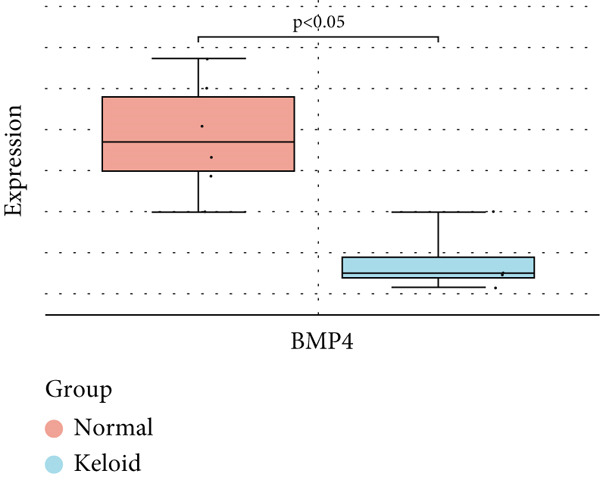
(a)

**Figure:**
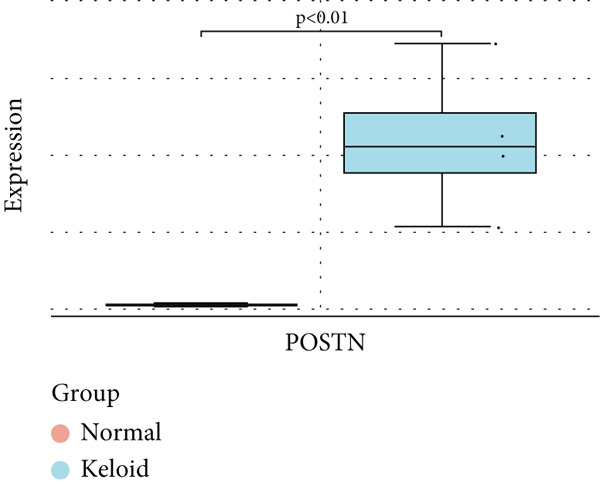
(b)

**Figure:**
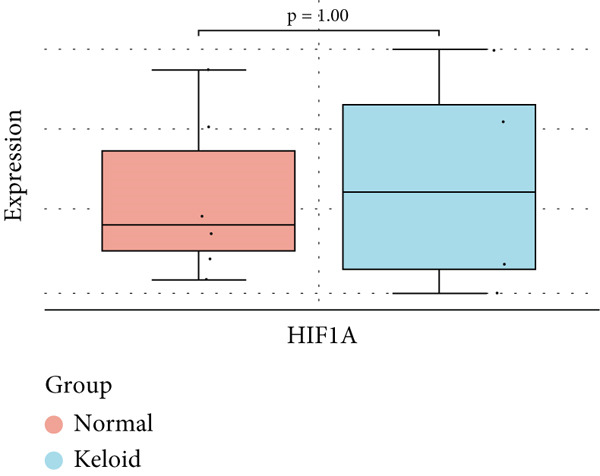
(c)

**Figure:**
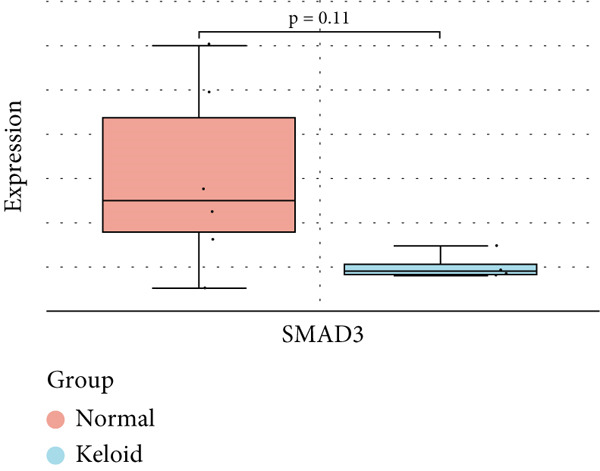
(d)

**Figure:**
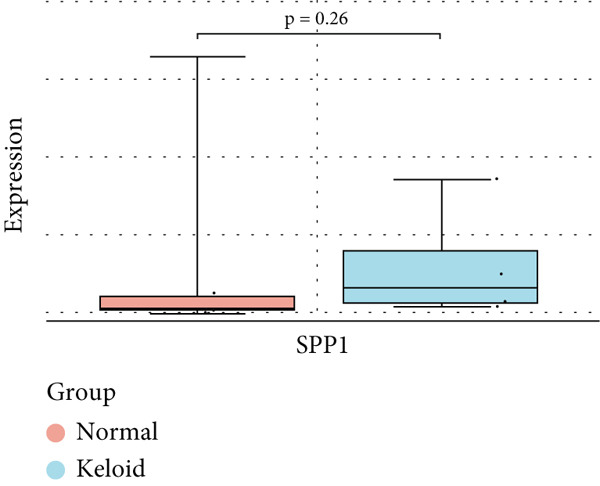
(e)

**Figure:**
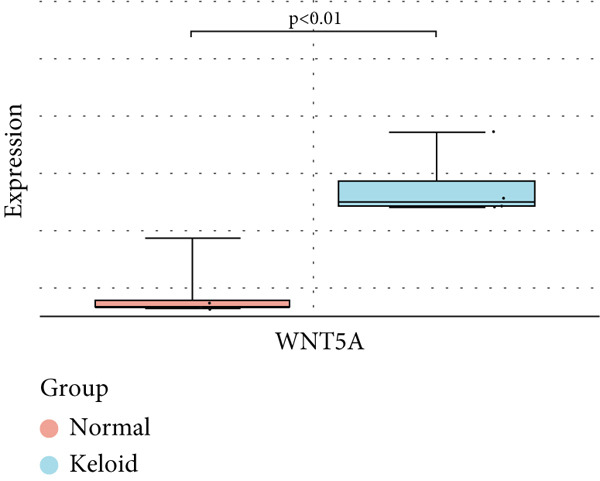
(f)

Figure 10(a–f) ROC curve of six hub genes.

**Figure:**
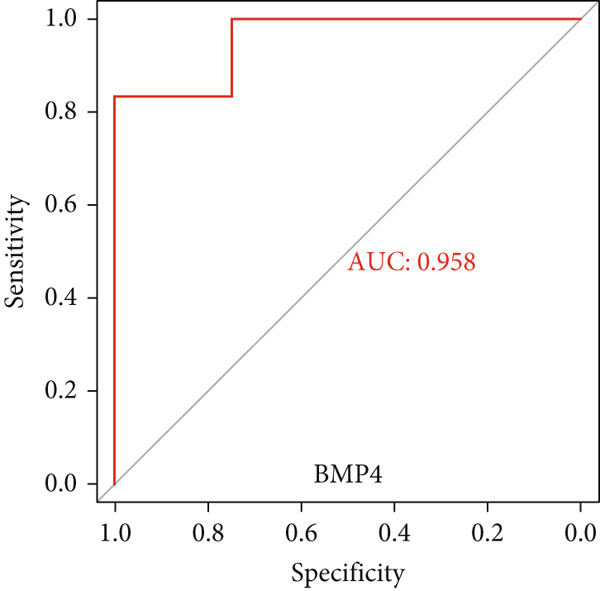
(a)

**Figure:**
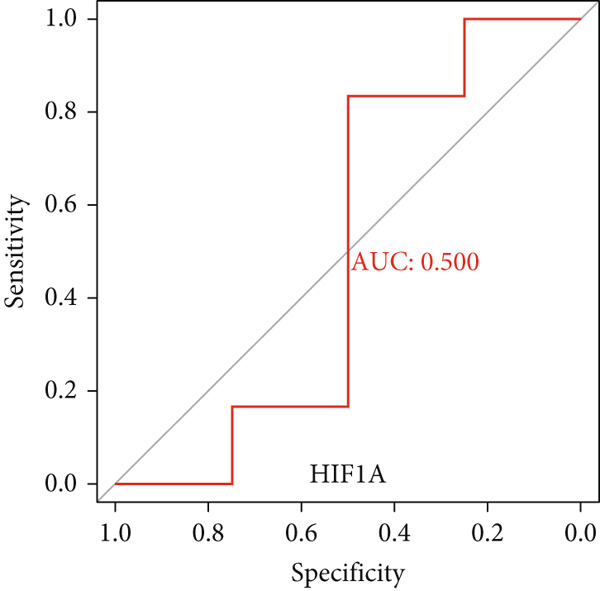
(b)

**Figure:**
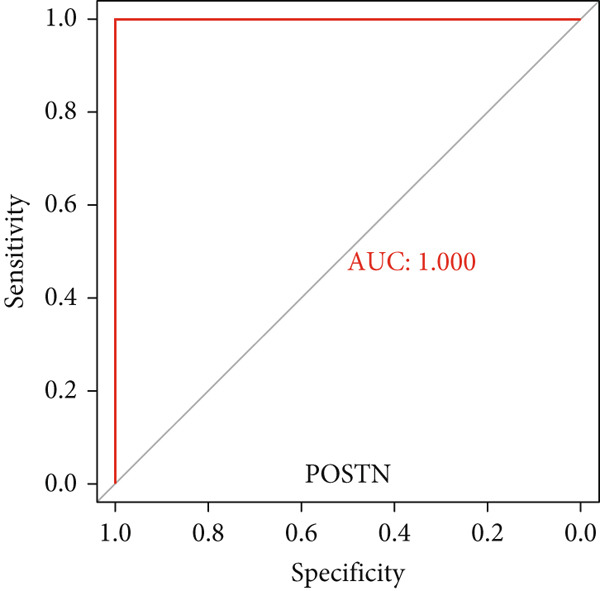
(c)

**Figure:**
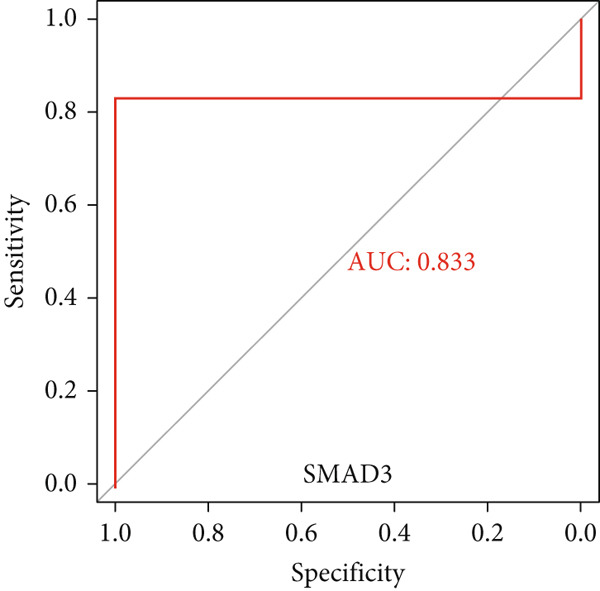
(d)

**Figure:**
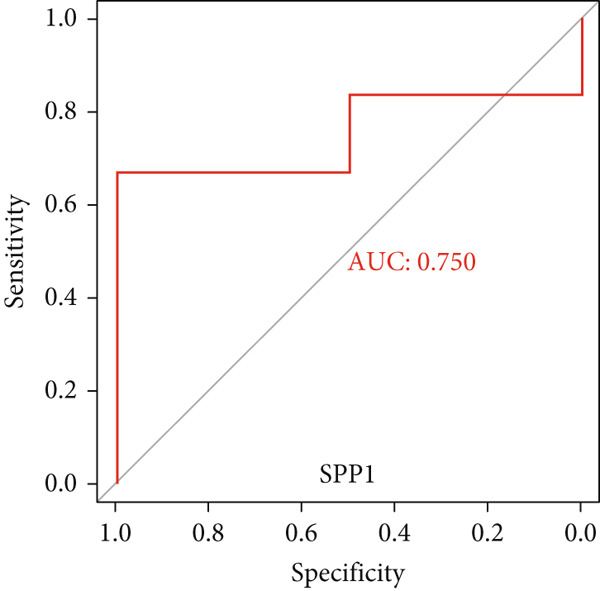
(e)

**Figure:**
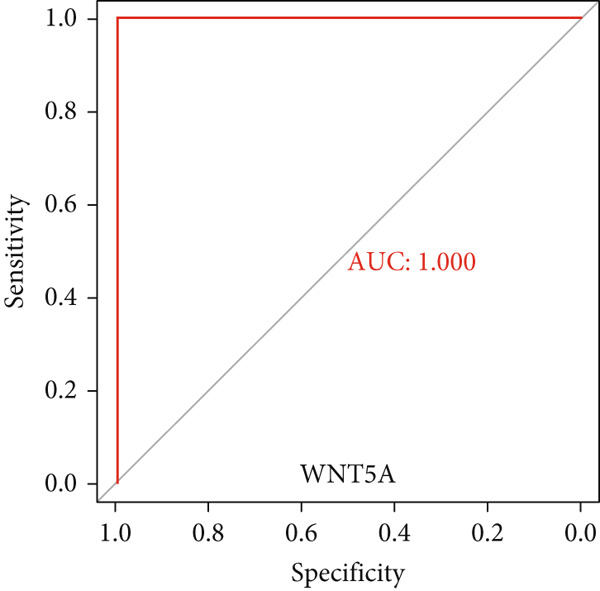
(f)

### 3.7. Results of Gene–miRNA and Gene–TF Interaction Networks

**Figure 11:**
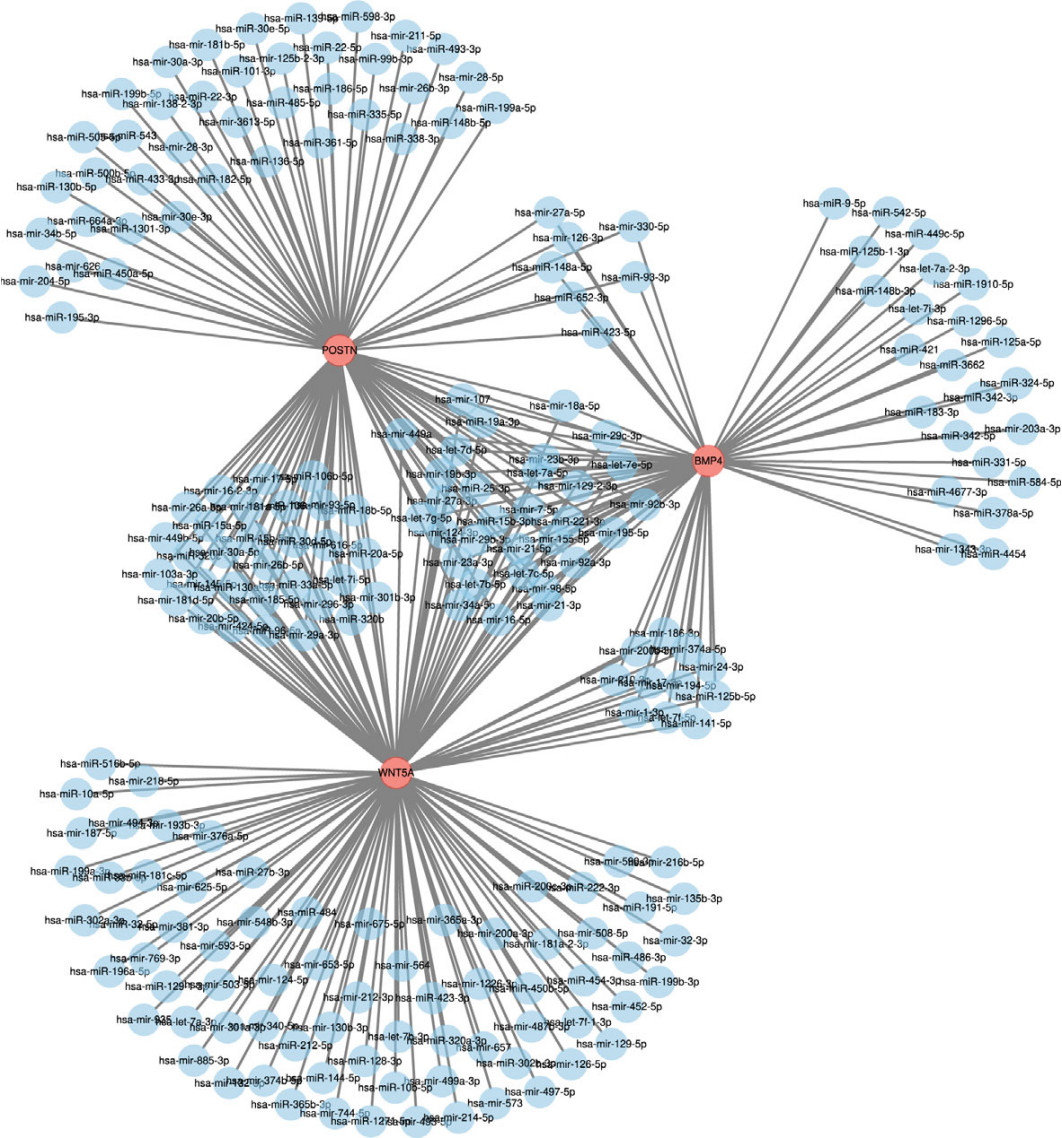
Interactions among three hub genes and miRNAs.

**Figure 12:**
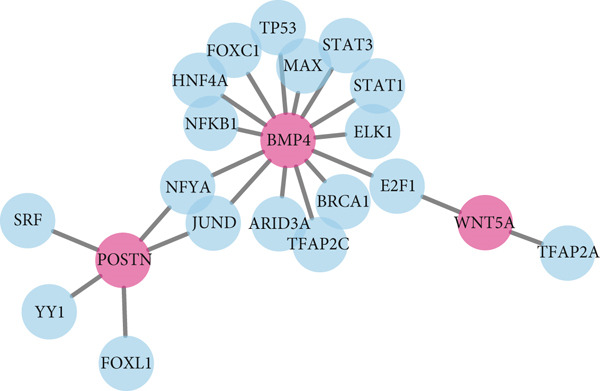
Interactions among three hub genes and transcription factors.

Hub genes and miRNA or TF interaction networks were generated using NetworkAnalyst. The gene–miRNA interaction network for the hub genes BMP4, POSTN, and WNT5A was constructed (Figure 11). The miRNA‐target gene interaction network revealed that POSTN, BMP4, and WNT5A are regulated by a large number of miRNAs, with POSTN being the most frequently targeted gene (> 70 miRNAs). Notably, several miRNAs, such as hsa‐mir‐27a‐5p, hsa‐mir‐330b‐5p, and hsa‐mir‐141‐5p, simultaneously target multiple key genes, suggesting coordinated regulatory mechanisms in keloid pathogenesis. This integrated regulatory network provides novel insights into the molecular basis of keloid formation and identifies potential therapeutic targets for future intervention. Subsequently, the BMP4, POSTN, and WNT5A genes–TF network was built, and the hub genes–TF interaction network was generated (Figure 12). The TF regulatory network shows that BMP4 is the gene with the greatest degree of regulation, being targeted by 14 different TFs including TP53 and STAT3, indicating its core role in integrating multiple signaling pathways. In contrast, POSTN is primarily regulated by factors such as SRF and JUND. However, WNT5A is regulated by TFAP2A and E2F1. This comprehensive regulatory framework provides a mechanistic basis for understanding how dysregulation of upstream transcriptional programs leads to the abnormal expression of fibrotic markers.

### 3.8. DSigDB Database for Drug–Gene Target Identification

To investigate potential therapeutic strategies for keloid fibroblasts, we employed the DSigDB database, applying stringent screening criteria. Specifically, our analysis focused on compounds with an *a* *d* *j*.*p*.*V* *a* *l* below 0.01, applying a rigorous statistical threshold to identify the most promising drug candidates. The Top 10 relevant drugs are listed, as shown in Table 1: paricalcitol, phosphine, butanoate, mitomycin C, paclitaxel, iloprost, EDTA, cytarabine, octreotide, and HMN‐176. This rigorous statistical threshold enabled the identification of two promising candidate agents: paricalcitol and phosphine. Both compounds are significantly associated with the BMP4 and POSTN genes.

**Table 1:** Candidate drug predicted using DSigDB.

**Drug names**	**p** **value**	**Adjusted** **p** **value**	**Genes**
Paricalcitol CTD 00003033	≤ 0.001	0.002	BMP4 and POSTN
Phosphine CTD 00006547	≤ 0.001	0.002	BMP4 and POSTN
Butanoate CTD 00005796	≤ 0.001	0.015	BMP4 and WNT5A
Mitomycin C CTD 00007136	0.001	0.021	BMP4 and WNT5A
Paclitaxel CTD 00007144	0.002	0.039	BMP4 and POSTN
Iloprost CTD 00007112	0.002	0.039	BMP4
EDTA CTD 00005888	0.003	0.039	BMP4
Cytarabine CTD 00005743	0.003	0.039	BMP4 and POSTN
Octreotide CTD 00007059	0.003	0.047	BMP4
HMN‐176 CTD 00004250	0.005	0.051	POSTN

## 4. Discussion

Keloids are a condition characterized by the abnormal proliferation of skin connective tissues, leading to the excessive accumulation of collagen fibers in the dermis [21, 22]. As the predominant cell type within keloid tissue, fibroblasts play a critical role in the initiation and progression of keloid formation [23]. Some research indicates that keloid fibroblasts demonstrate increased production of mature collagen, TGF‐*β*, HIF1*α*, and *α*‐SMA relative to their counterparts in healthy dermis, highlighting the central reticular zone as a key site of ECM deposition and a potential driver of lesion progression through radial tissue expansion [24]. Epithelial–mesenchymal transition (EMT) contributes to keloid pathogenesis by facilitating the transformation of epithelial‐derived cells into a profibrotic phenotype, thereby promoting ECM deposition and fibroblast proliferation [25]. Current treatments for keloids primarily include pharmacological and surgical approaches [26–30]. For example, triamcinolone acetonide (TAC; Kenalog), a corticosteroid injection, remains the first‐line treatment for keloids [31]. Corticosteroids can modulate several key pathways involved in the formation and development of keloids by reducing the inflammatory response during wound healing, which in turn affects multiple core pathways in the growth and development of keloids [32–34]. Additionally, corticosteroids inhibit the synthesis of collagen and glycosaminoglycans, thereby suppressing fibroblast development and promoting collagen degradation [35]. This therapy can be used as a primary treatment for keloids or in conjunction with surgical or laser therapy [36]. Combined medical–phototherapy treatments, such as verapamil, ultraviolet A1 phototherapy in the wavelength range of 340–400 nm, and angiotensin‐converting enzyme inhibitors (ACEIs), as well as the application of new technologies, have been shown to significantly inhibit fibroblast proliferation in keloids [37, 38]. Cheng et al. found that the major fibroblast subsets in keloids exhibit a mechanoresponse phenotype, characterized by enhanced mechanotransduction and migration activity. This may be a key driver of the aggressive growth behavior observed in keloids [39]. The choice of treatment for keloids depends on the size and number of the lesions. Small and isolated keloids can be managed through surgical excision and isotope therapy (e.g., radiation therapy). For patients with large and multiple keloids, surgical treatment is often employed to reduce the quantity of keloids [36, 40]. Keloids are a group of proliferative dermatoses characterized by the overproduction of fibroblasts, manifesting as benign skin lesions that result in excessive deposition of collagen fibrillar proteins following injuries of varying severity [41]. The primary cells responsible for collagen fibril deposition are fibroblasts, whose gene expression is influenced by a series of regulatory factors, including cytokines, growth factors, and noncoding RNAs [42–44].

Our integrative bioinformatics analysis of the GSE145725 dataset identified 496 DEGs between keloid fibroblasts and normal tissues, with functional enrichment highlighting key roles for pathways involved in FA, Wnt signaling, and transcriptional misregulation in cancer. Notably, despite the established centrality of the TGF‐*β* pathway in fibrosis, our KEGG analysis surprisingly indicated that downregulated genes were enriched in the TGF‐*β* signaling pathway—a finding that may reflect compensatory feedback mechanisms or heterogeneity in fibroblast subpopulations. Nevertheless, PPI network analysis and subsequent validation using the independent GSE158395 dataset consistently identified BMP4, WNT5A, and POSTN as top hub genes significantly upregulated in keloid tissues. These three genes, while not the most traditionally emphasized in keloid pathogenesis, may represent novel convergent nodes linking mechanical signaling, ECM remodeling, and inflammatory activation in keloid fibroblasts.

BMP4 is a protein‐coding gene. It is expressed by endothelial cells (ECs) in response to hypoxia and promotes vascular smooth muscle cell (SMC) proliferation, which can induce angiogenesis, EC proliferation, and migration [45]. This gene encodes a secreted ligand for the TGF‐*β* superfamily of proteins. Ligands of this family bind to various TGF‐*β* receptors, leading to the recruitment and activation of SMAD family TFs, which regulate gene expression [46]. Elevated BMP4 may paradoxically contribute to fibroblast activation by modulating SMAD signaling, potentially synergizing with or fine‐tuning canonical TGF‐*β*/SMAD activity. Some studies [47] indicate that BMP4 is closely related to the regrowth of hair follicles, and strategies for regenerating hair follicles could ultimately impact the development and treatment of keloids. This pathway is a major regulator of fibroblast and myofibroblast activation, driving excessive collagen synthesis and ECM deposition. Continued activation of TGF‐*β*/SMAD signaling creates a profibrotic feedback loop that maintains fibroblast hyperactivity, leading to the chronic, nonregressing nature of keloid lesions.

Our analysis identified HIF1*α* as a central hub gene in the regulatory network, which is consistent with previous findings by Kang et al. [10], who also highlighted HIF1*α*′s role in regulating collagen production in keloids; however, there is no significant difference in the validation dataset, and it can be used as a comprehensive predictor of keloid fibroblasts.

POSTN is a gene predominantly found in bone tissues and is mainly distributed in the ECM. The expression level of POSTN is closely related to the development of various diseases [48–50] which is often associated with delayed wound healing, delayed regeneration of epithelial tissue, and a reduction in keratinocytes [51]. Additionally, POSTN can promote tissue healing by enhancing the activation, proliferation, differentiation, and contraction of fibroblasts [52]. Previous studies have already indicated the dysregulation of POSTN in keloids, with Xu et al. highlighting its role as a crucial node in the pathogenesis of these lesions [53]. This reinforces the potential role of POSTN in keloid development and supports its consideration as a candidate therapeutic target. In the present study, POSTN was found to be significantly upregulated in keloids. Its overexpression in keloids is consistent with its role in stabilizing collagen fibrils and facilitating ECM cross‐linking—processes that contribute to the rigidity and persistence of keloid lesions. However, experimental validation of POSTN in keloids has not yet been reported, and further studies are needed to verify this.

WNT5A plays a role in cellular pathways and inflammatory processes [54]. This gene encodes both long‐chain and short‐chain WNT5A proteins, which have different functions depending on the methylation status of the WNT5A gene promoter [55]. WNT5A contributes to the abnormal activation of fibroblasts and EMT‐like phenotypic changes in neighboring Kupffer cells (KCs) through the IL‐6/JAK/STAT3 pathway, which can induce EMT‐like transitions in epithelial and stromal cells, thereby increasing the pool of activated fibroblasts and promoting ECM overproduction [25, 56]. In our dataset, the upregulation of WNT5A aligns with the observed enrichment in FA and cytoskeletal reorganization pathways, suggesting a role in enhancing fibroblast motility. Thus, WNT5A may serve as a critical link between inflammatory cues and structural remodeling in keloid progression, and the WNT5A signaling pathway may help develop new strategies for early intervention in keloid formation [57, 58].

The purpose of this research was to investigate the mechanisms underlying keloid development and to explore potential therapeutic strategies. Our results demonstrate that there are genes with significant variations in expression levels between keloid and normal skin, which further underscores the importance of these genes in the pathogenesis of keloid. We found that the expression levels of BMP4, POSTN, and WNT5A were significantly higher in keloid samples compared to control samples, as identified through differential gene screening and validated using an independent dataset. However, specific biomarkers for the diagnosis and treatment of keloids have not yet been established.

GO and KEGG enrichment analyses of the DEGs revealed that signaling pathways such as FA, mineral absorption, renin secretion, and the TGF‐*β* signaling pathway are closely related to the formation of keloids. FAs are biological structures involved in cellular processes such as migration, polarization, and cancer formation [59–61]. The TGF‐*β*/SMAD signaling pathway is a key pathway associated with collagen synthesis in fibroblasts. Persistent activation of the TGF‐*β*/SMAD signaling pathway can lead to the overstimulation of fibroblasts, resulting in excessive collagen production and deposition, which is a characteristic of keloid formation [20]. It is important to note that the PPI network in this study was inferred based on RNA‐sequencing data of DEGs. While changes in mRNA expression often correlate with changes in protein abundance, this is not always the case due to posttranscriptional and posttranslational regulatory mechanisms. Therefore, the observed interactions in the network represent hypothesized protein‐level changes based on transcriptomic data. Future studies involving direct proteomic analysis and validation of key interactions are warranted to confirm the actual protein expression levels and interactions within keloid fibroblasts.

We propose a potential cooperative mechanism: Hypoxia or injury‐induced BMP4 may initiate mesenchymal cell activation and angiogenesis; WNT5A, potentially upregulated in response to inflammation or mechanical stress, drives fibroblast migration and EMT‐like plasticity via IL‐6/STAT3; and POSTN, secreted by activated fibroblasts, reinforces ECM deposition and stabilizes the fibrotic niche through integrin signaling. This triad may form a self‐sustaining loop that promotes radial expansion and resistance to regression in keloids. It is important to acknowledge that our analysis is based on transcriptomic data, and posttranscriptional regulation (e.g., miRNA, methylation, and protein modification) may affect the actual functional output of these genes.

Through screening, we identified paricalcitol and phosphine as the most promising drug candidates for the treatment of keloids. Paricalcitol is a selective vitamin D receptor activator. Some research has shown that paricalcitol is involved in chronic inflammatory processes and promotes granuloma stabilization and maturation by regulating immune pathways [62]. Phosphine (tris(2‐carboxyethyl) phosphine) is a very effective thiol reducing agent, widely used in protein chemistry and proteomics research as a quantitative reducing agent for the reduction of disulfide bonds while preserving other functional groups in the proteins [63]. Some research indicates that phosphine may induce hepatotoxicity by disrupting the HPT and the gut–liver axis, which can lead to liver inflammation and oxidative stress [64]. Therefore, paricalcitol and phosphine contribute to advancing precision medicine and make an outstanding contribution to the development of new drugs for keloids.

In conclusion, our study identifies BMP4, WNT5A, and POSTN as novel and consistently upregulated hub genes in keloids, potentially acting at the nexus of mechanosignaling, inflammation, and matrix remodeling. These findings not only expand the molecular landscape of keloid pathogenesis but also highlight new candidates for diagnostic biomarkers or targeted therapies. Future studies focusing on the crosstalk between these genes and their downstream effectors may uncover actionable pathways for interrupting the fibrotic cascade in keloids.

## 5. Conclusion

This study has identified three hub genes: BMP4, POSTN, and WNT5A. These genes are expected to serve as potential biomarkers and may provide valuable insights that will contribute to the development of novel therapeutic strategies for keloid fibroblasts. However, the method of selecting hub genes through bioinformatics analysis still has certain limitations; further molecular biology experiments and animal model studies are required to elucidate the specific mechanisms of keloid formation and development.

## Ethics Statement

The authors have nothing to report.

## Disclosure

All authors contributed to the manuscript, have approved the final version, and agreed to be accountable for the content and conclusions presented in this article.

## Conflicts of Interest

The authors declare no conflicts of interest.

## Author Contributions

B.Z. and W.Z. were responsible for the conception and design of the study, data acquisition, and bioinformatics analysis. W.Y. and Xin.L. performed the enrichment analyses. T.Y. and H.C. constructed networks using Cytoscape software. Xia.L. supervised the project, provided critical feedback, and helped shape the research, analysis, and manuscript. B.Z. and W.Z. contributed equally to this work and are considered co‐first authors, sharing first authorship.

## Funding

This study was funded by the Research Fund of Anhui Institute of Translational Medicine (2023zhyx‐C57), the Postgraduate Innovation Research and Practice Program of Anhui Medical University (YJS20230120), and the Horizontal Project of the First Affiliated Hospital of Anhui Medical University (20240013).

## Data Availability

All the data generated or analyzed during this study are included in this published article.
